# Diversity and Network Structure of Culicidae Associated with Two Bromeliad Species from Northwestern Argentina

**DOI:** 10.3390/insects17080865

**Published:** 2026-08-20

**Authors:** María Julia Dantur-Juri, Jonathan Liria-Salazar, Gabriela Cecilia Flores, Luciana Contreras, Gabriela María Silenzi-Usandivaras, Paul Leonardo Duque, Raúl Ernesto Campos, Ximena Pérez-Otáñez

**Affiliations:** 1 Unidad Ejecutora Lillo (CONICET-Fundación Miguel Lillo), San Miguel de Tucumán 4000, Tucumán, Argentina; gaflores363@gmail.com (G.C.F.); polduquebiologo@gmail.com (P.L.D.); 2 Instituto de Genética y Microbiología, Fundación Miguel Lillo, San Miguel de Tucumán 4000, Tucumán, Argentina; lcontreras@lillo.org.ar (L.C.); gmsilenzi@lillo.org.ar (G.M.S.-U.); 3 Grupo de Investigación en Población y Ambiente, Universidad Regional Amazónica Ikiam, Napo 150101, Ecuador; jonathan.liria@gmail.com; 4 Instituto de Limnología “Raúl A. Ringuelet”, Universidad Nacional de La Plata—CONICET, La Plata 1900, Buenos Aires, Argentina; 5 Facultad de Medicina Veterinaria y Agronomía, Universidad UTE, Quito 170129, Ecuador; ximena.perez@ute.edu.ec; 6 Grupo de Investigación CABSAC, Facultad de Medicina Veterinaria y Agronomía, Universidad UTE, Quito 170129, Ecuador

**Keywords:** phytotelmata, interaction network structure, Culicidae, Argentina

## Abstract

Phytotelmata are natural water-holding microhabitats that support diverse aquatic communities, including immature mosquitoes. We analyzed the diversity, composition, and interaction network structure of Culicidae associated with two bromeliad species, *Aechmea distichantha* and *Vriesea friburgensis*, in two localities of the Yungas ecoregion in northwestern Argentina: La Florida Provincial Reserve (LFPR) (Tucumán) and Isla de Cañas (Salta). Immature mosquitoes were sampled during 2017–2018, and community structure was evaluated using Hill numbers, nonparametric richness estimators, bipartite network analysis, and canonical correspondence analysis (CCA). A total of 12 mosquito species were identified, with richness ranging from 5 to 9 species depending on bromeliad species and locality, with the highest richness recorded in *V. friburgensis* in LFPR. Sampling completeness ranged from 96.8% to 100%. Bipartite network analysis showed moderate connectance (0.50), low modularity (Q = 0.188), intermediate nestedness (NODF = 46.94), and low global specialization (H2′ = 0.273), suggesting a relatively generalized interaction structure. *Culex coronator* and *Cx. imitator* dominated the assemblage, while other species exhibited more restricted associations. The CCA indicated that species composition was mainly associated with bromeliad height above ground, internal water temperature, sediment, and pH. These results highlight the importance of microhabitat heterogeneity in structuring mosquito assemblages in Neotropical phytotelmata.

## Simple Summary

Bromeliads store rainwater in small natural reservoirs that create aquatic microhabitats for many organisms, including mosquitoes in their immature stages. These plants can host a wide variety of mosquito species, some of ecological importance and others relevant to public health because they may act as disease vectors. In this study, we analyzed the relationships between mosquito communities and bromeliad species in the Yungas forests of northwestern Argentina. We found that these communities are ecologically complex and include both widespread species and others with more restricted distributions. Mosquito communities differed not only in the number and abundance of species but also in how species shared and used bromeliad microhabitats. Our findings improve understanding of mosquito diversity and ecology in natural plant-associated habitats and provide useful information for future studies in environments with different levels of conservation and environmental change.

## 1. Introduction

The Bromeliaceae family comprises approximately 80 genera, encompassing a total of 3740 species distributed worldwide [1,2]. Although primarily Neotropical, some species within this plant group extend their range into the southern United States [3,4,5]. Among the most diverse genera are *Tillandsia* sp. (730 described species), *Pitcairnia* sp. (407 described species), *Aechmea* sp. (284 described species), *Vriesea* sp. (261 described species), and *Puya* sp. (225 described species) [6].

Bromeliads have developed adaptations to function as phytotelmata, resulting in plant structural modifications such as the formation of water-holding structures [5]. Water accumulates within the axils of the inner leaves and in the central cavity known as the water tank [7,8], serving as an aquatic microecosystem [9]. Such structures harbor a high diversity of organisms, including microorganisms, invertebrates, and, in some cases, small vertebrates [2,10].

Mosquitoes constitute one of the groups most closely associated with bromeliads [11,12]. In fact, the presence of immature stages of more than 200 species has been recorded in these microhabitats, representing approximately 20% of total Culicidae diversity [13]. Among them, the genera most frequently associated with bromeliads include *Culex* (*Microculex*), *Aedes* (*Howardina*), *Wyeomyia*, and *Anopheles* (*Kerteszia*) [14]. In addition to their ecological significance, phytotelmata are of public health importance, as they serve as breeding sites for mosquitoes that act as vectors of human and animal pathogens [9]. These include *An. cruzii* Dyar & Knab, 1908 and *An. bellator* Dyar & Knab, 1906 (subgenus *Kerteszia*), linked to the so-called “bromeliad malaria”, as well as *Haemagogus* (*Conopostegus*) *leucocelaenus* (Dyar & Shannon, 1924), a vector of sylvatic yellow fever, and medically important invasive species such as *Ae.* (*Stegomyia*) *aegypti* (Linnaeus, 1762) and *Ae.* (*Stg.*) *albopictus* (Skuse, 1894), associated with the transmission of arboviruses [9,15,16,17].

In Argentina, the Bromeliaceae family is represented by 15 genera and approximately 100 species [18]. Among the most prominent genera are *Aechmea*, *Vriesea*, *Tillandsia*, and *Dyckia* spp., including species such as *Aechmea distichantha* Lem, *Vriesea friburgensis* var. *tucumanensis* (Mez) L.B. Sm., *Tillandsia australis* Mez, *Tillandsia aeranthos* (Loisel.) L.B. Sm., and *Aechmea miniata* var. *discolor* Beer ex Baker [17,19,20].

*Aechmea distichantha* stands out due to its wide geographic distribution, occurring in at least nine provinces, and for its high productivity, contributing to a high abundance of mosquito larvae. Consequently, *Cx.* (*Mcx.*) *imitator* Theobald, 1903, emerges as the species most frequently recorded [19]. Furthermore, the occurrence of other culicids has been reported in this plant species, such as *Cx. davisi* Kumm, 1933, *Cx. fernandezi* (Casal, García & Cavalieri, 1966), *Wy.* (*Phoniomyia*) *muehlensi* (Petrocchi, 1927), *Wy.* (*Miamyia*) *limai* Lane & Cerqueira, 1942, *Wy.* (*Pho.*) *quasilongirostris* (Theobald, 1907), *Toxorhynchites* (*Lynchiella*) *haemorrhoidalis separatus* (Lynch Arribálzaga, 1891), *Tx.* (*Lyn.*) *guadeloupensis* (Dyar & Knab, 1906), *Tx.* (*Lyn.*) *solstitialis* (Lutz, 1904), *Tx.* (*Lyn.*) *bambusicola* (Lutz & Neiva, 1913), and *Trichoprosopon pallidiventer* (Lutz, 1905). Likewise, the occurrence of vectors of public health relevance has been reported, including *Ae*. *aegypti* and *Cx*. *quinquefasciatus* Say, 1823, particularly in transitional environments between native forests and urbanized areas [16,17,19,21,22,23].

Regarding *V*. *friburgensis*, it constitutes a key species in the Yungas ecoregion of northwestern Argentina, where it functions as a structural epiphyte in the montane forest canopy [17]. Moreover, this species has been identified as a host plant for a wide array of mosquito species, including *Cx. hedys* Root, *Cx*. *imitator*, *Cx. neglectus* Lutz, *Cx. albipes* Lutz, *Cx*. *davisi*, *Cx. pleuristriatus* Theobald, *Wy. incaudata* Root, *Tx*. *solstitialis*, and *Ae*. *aegypti* [15,17].

The association between bromeliads and mosquitoes is modulated by environmental variables through a cascading effect, where macroclimate conditions the physical and chemical characteristics of the microhabitat [1]. Precipitation increases water volume in phytotelmata, while high temperatures reduce it by evaporation; in general, a greater volume is associated with higher richness and abundance of mosquito species [1,3].

The volume also regulates chemical parameters such as pH, which tends to increase with rainfall and constitutes a key factor in species distribution, as observed for *An*. *cruzii*, which is associated with less acidic conditions [1].

In urban environments, higher water temperatures may accelerate larval development, favoring the proliferation of invasive species such as *Ae*. *albopictus* [24]. Landscape factors, such as elevation, also influence diversity, with higher richness levels in lowland areas [25]. In this regard, seasonal variations in temperature and humidity have been identified as key determinants of colonization and survival cycles in the phytotelmata of *A*. *distichantha* [26].

To understand how environmentally induced microhabitat variability shapes mosquito assemblages, the present research aimed to analyze the influence of *A*. *distichantha* and *V*. *friburgensis* microhabitats on the structure, composition, and diversity of the associated mosquito communities, as well as to explore plant–mosquito interactions in two areas of the Yungas region of northwestern Argentina.

## 2. Materials and Methods

### 2.1. Sampling Sites

**Figure 1:**
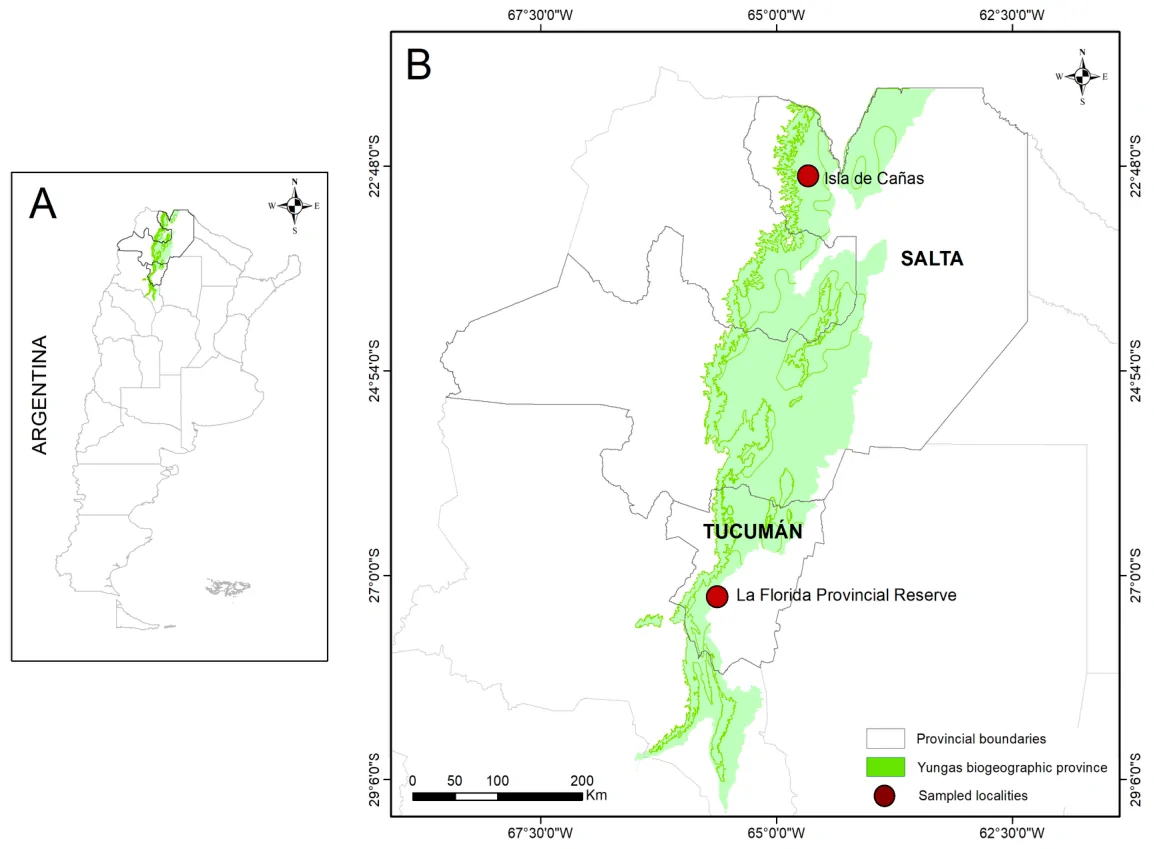
(**A**) Location of the study area in Argentina. (**B**) Sampled localities (Isla de Cañas, Salta and La Florida Provincial Reserve, Tucumán) in the Yungas biogeographic province.

This study was carried out in two forest patches within the Yungas of northwestern Argentina, situated within the provinces of Salta (northern patch) and Tucumán (meridional patch). In Salta Province, Isla de Cañas was the selected locality (22°53′58.4” S, 64°39′55.5” W), situated in a transitional area between pedemontane and montane rainforests. In Tucumán, the selected locality was the La Florida Provincial Reserve (LFPR) (27°13′07.9” S, 65°37′44.2” W), located within the pedemontane forest (Figure 1). Both localities represent two distinct biogeographic localities of the Yungas characterized by significant ecological differences, particularly regarding latitudinal and altitudinal gradients, precipitation regimes, and phytotelmic microhabitat availability.

The sampling site in Isla de Cañas is situated in the northern patch of the Yungas pedemontane rainforest (400–700 m a.s.l.), within a forest type locally known as “palo blanco” and “palo amarillo” (*Calycophyllum multiflorum* and *Phyllostylon rhamnoides*, respectively). The climate is subtropical and influenced by the Sierra de Santa Victoria, generating a warm, humid environment with annual precipitation ranging between 800–1000 mm. More than 90% of this rainfall is concentrated in the summer period (November–March) [27,28].

The meridional patch, located in LFPR, encompasses a pedemontane forest (550–900 m a.s.l.), characterized by tipa (*Tipuana tipu*) and pacará (*Enterolobium contortisiliquum*). Other prominent tree species present in this forest are Palo de San Antonio (*Rapanea laetevirens*), cebil (*Anadenanthera colubrina*), large specimens of laurel (*Cinnamomum porphyrium*), walnut (*Juglans australis*), and horco molle (*Blepharocalyx salicifolius*), with an understory dominated by the fern *Pteris deflexa*, while tree trunks are typically covered by mosses and epiphytic assemblages of ferns, bromeliads, and Piperaceae. The climate is humid subtropical, featuring hot, high-rainfall summers (exceeding 2500 mm annually) and mild winters characterized by frequent fog [29].

### 2.2. Sampling of Mosquito Immature Stages

**Figure 2:**
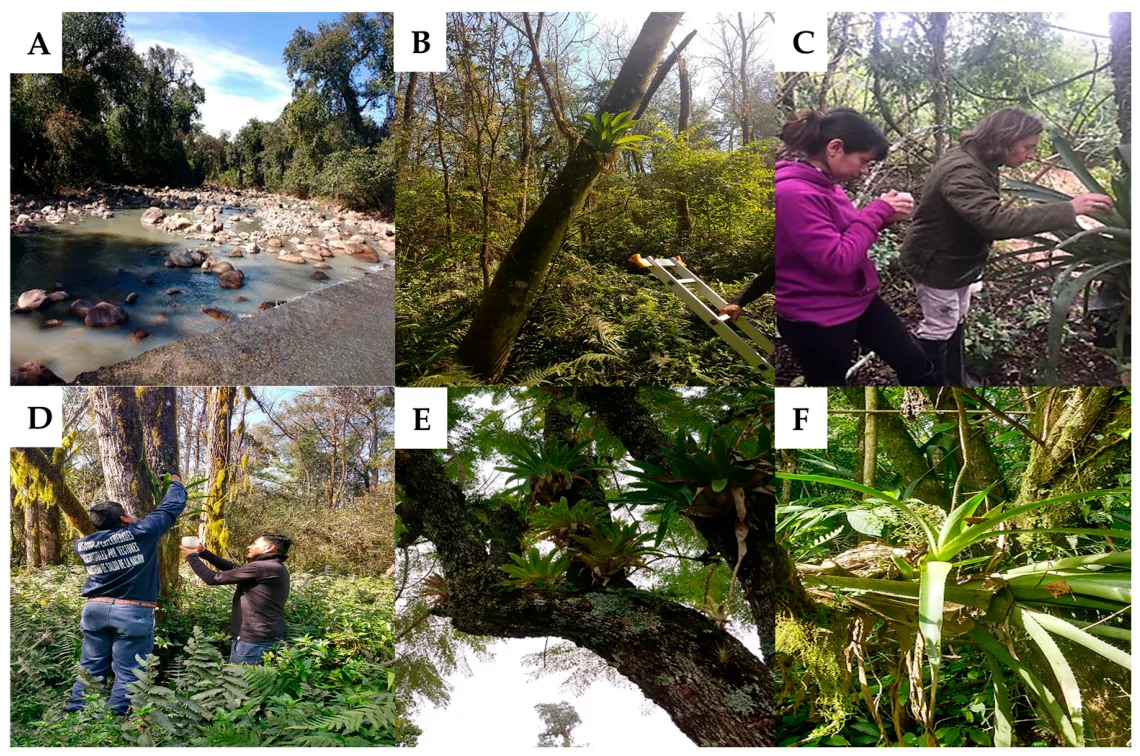
(**A**) Landscape view of the Yungas biogeographic province (Salta). (**B**) Epiphytic bromeliad growing on the mid-trunk of a tree. (**C**,**D**) Collection of immature larvae from bromeliad phytotelmata. (**E**) *Vriesea friburgensis* growing on the upper branches of a host tree. (**F**) *Aechmea distichantha* growing at the base of tree trunks.

In both localities, immature stages were collected during three consecutive days across two seasonal periods: April, May, and June (first semester), and August, September, and October (second semester). During each sampling month within these periods, collections were conducted for three consecutive days in 2018 and 2019. Bromeliads were randomly selected from plants accessible to the sampler in both localities. Larvae and pupae were collected using a 50 mL plastic pipette, transferring the contents to 500 mL PVC containers for transportation to the laboratory (Figure 2A–F). The containers with the immature mosquito samples (larvae and pupae) were processed at the laboratory of the Unidad Ejecutora Lillo (CONICET-Fundación Miguel Lillo). A proportion of the larvae were reared individually in 5 cm high plastic cups, each covered with voile fabric, until they reached the fourth instar. Subsequently, they were killed and preserved in 90% alcohol for taxonomic identification [30]. The remaining individuals were reared to adulthood; larval and pupal exuviae were associated with each adult mosquito specimen following standard procedures for ecological and taxonomic studies of mosquitoes [31]. Adult specimens were identified using the dichotomous keys [30,32,33].

The taxonomic identification of the bromeliad species collected in northwestern Argentina was conducted by the botanical specialist, Dr. Hugo Ayarde, from Fundación Miguel Lillo, Tucumán, Argentina (FML). The adult mosquito specimens were pinned following standardized entomological protocols and are deposited in the entomological collection of the Instituto-Fundación Miguel Lillo (IMLA), Fundación Miguel Lillo, Tucumán, Argentina.

During each sampling event, data on the geographic collection locality, date, phytogeographic region, ambient temperature, and relative humidity were recorded. In addition, the structural variables of each bromeliad were measured, including basal diameter, total length, number of leaves, leaf width, and height above ground. The physicochemical variables of the aquatic microhabitat were also recorded: internal water temperature, external ambient temperature, pH, volume of the water contained within the bromeliad, and mass of sediment present in the water.

### 2.3. Data Analysis

The abundance of Culicidae species was estimated based on number of specimens per species, and subsequent analyses were performed at different scales according to their specific objectives. Abundance, diversity, and evenness were described per sampling period to reflect the temporal variation in the communities. Conversely, species accumulation curves, bipartite networks, and Canonical Correspondence Analysis (CCA) were constructed using matrices aggregated by locality and bromeliad species to assess global patterns of richness, interaction structure, and associations with environmental variables while preventing excessive dataset subdivision.

#### 2.3.1. Diversity and Evenness Analysis

The structure, composition, and diversity of Culicidae communities associated with bromeliads were assessed based on both host plant identity and geographic sampling site. Diversity was characterized through the calculation of Hill numbers, where q = 0 represents species richness, q = 1 represents the diversity of common species, and q = 2 represents the diversity of very common species. This approach allowed diversity to be expressed as the effective number of species and facilitated ecologically interpretable comparisons between assemblages with different abundance distributions [34]. Additionally, evenness was estimated using the H1/H0 ratio, applied as a measure of uniformity across species abundance distribution.

#### 2.3.2. Nonparametric Richness Estimators

The potential species richness of Culicidae associated with bromeliads was estimated through nonparametric richness estimators (ACE, ICE, Chao 1, and Jackknife 1) employing EstimateS 9.1.0 software [35], which enabled the comparison of observed and expected richness and the assessment of sampling completeness based on incidence or abundance data [35]. Furthermore, the trends of both accumulation and estimation curves were used to evaluate whether the sampling effort was sufficient to capture most of the richness present in each combination of locality and bromeliad species.

#### 2.3.3. Bipartite Network Analysis

Bipartite networks based on occurrence and abundance matrices were constructed to analyze associations between Culicidae and Bromeliaceae species; to this end, the bipartite package in R version 4.3.1 [36] was employed. Metrics were calculated to describe different aspects of network architecture, including connectance, nestedness, modularity, and specialization. These metrics are widely utilized to evaluate the degree of generalization, redundancy, and organization of ecological interactions in bipartite networks [37].

#### 2.3.4. Multivariate Analysis

Finally, the relationship between Culicidae species composition and microhabitat environmental variables was assessed by means of Canonical Correspondence Analysis (CCA) [38] using PAST software version 4.13 [39]. The explanatory variables included in this analysis were pH, water volume, sediment quantity, and bromeliad height above ground. Prior to analysis, the environmental variables were log-transformed to reduce distribution asymmetry and mitigate differences in scales between variables. Similarly, the Culicidae abundance matrix was subjected to a Hellinger transformation before the CCA to diminish the effect of highly abundant species and manage the high proportion of zeros in the data matrix, thus enhancing the representation of compositional patterns among samples [40].

## 3. Results

### 3.1. Abundance, Richness, Diversity, and Evenness

Immature stages of Culicidae belonging to 12 species, distributed within the Anophelinae and Culicinae subfamilies, were recorded in the phytotelmata of *A. distichantha* and *V. friburgensis*. The species composition varied among localities, bromeliad species, and sampling periods. The highest values of total abundance were recorded for *A*. *distichantha* (420 individuals) and *V*. *friburgensis* (418 individuals), both occurring in LFPR in 2017, whereas the minimum value corresponded to *A*. *distichantha* at Isla de Cañas in 2017 (209 individuals) (Table 1).

The most abundant species within the overall sample were *Cx*. *coronator* and *Cx*. *imitator*, occurring at both localities and in both bromeliad species, albeit with fluctuations in their relative abundance. In LFPR, *An*. *argyritarsis* and *Cx*. *chidesteri* also represented a significant proportion of the community, while species such as *Ae*. *aegypti*, *Cx*. *dolosus*, *Cx*. *fernandezi*, *Tx*. *guadalupensis*, and *Tx*. *bambusicola* exhibited a more restricted distribution.

In Isla de Cañas, along with *Cx*. *coronator* and *Cx*. *imitator*, the presence of *Wy*. *oblita*, *Wy*. sp., and *Cx*. *hepperi* was notable, especially in *V*. *friburgensis*. The observed species richness (q = 0, Hill N0) ranged from 5 to 9 species. The maximum value was recorded in *V*. *friburgensis* from LFPR during 2017 (H0 = 9), followed by the same bromeliad species in 2018 (H0 = 7), while in the remaining locality–bromeliad combinations, the richness was 5 species. Similarly, the diversity of the common species (H1) peaked in *V*. *friburgensis* in LFPR in 2017 (H1 = 5.89) and 2018 (H1 = 5.32), indicating that this bromeliad harbored the most diverse community in the study in terms of effective abundance. The diversity of the very common species (H2) exhibited its highest value in *A*. *distichantha* at Isla de Cañas in 2017 (H2 = 2.18), while the remaining values ranged between 1.26 and 1.92, reflecting varying degrees of diversity between localities and bromeliad species (Table 1).

The estimated evenness resulting from employing the H1/H0 ratio ranged between 0.52 and 0.84. The highest value was observed in *A*. *distichantha* in LFPR in 2017 (0.84), while the lowest was recorded in *A*. *distichantha* at Isla de Cañas in 2017 (0.52). Overall, these results revealed that *V*. *friburgensis* in LFPR presented the highest richness and effective diversity, whereas evenness and dominance fluctuated between localities and bromeliad species (Table 1).

### 3.2. Nonparametric Richness Estimators

**Figure 3:**
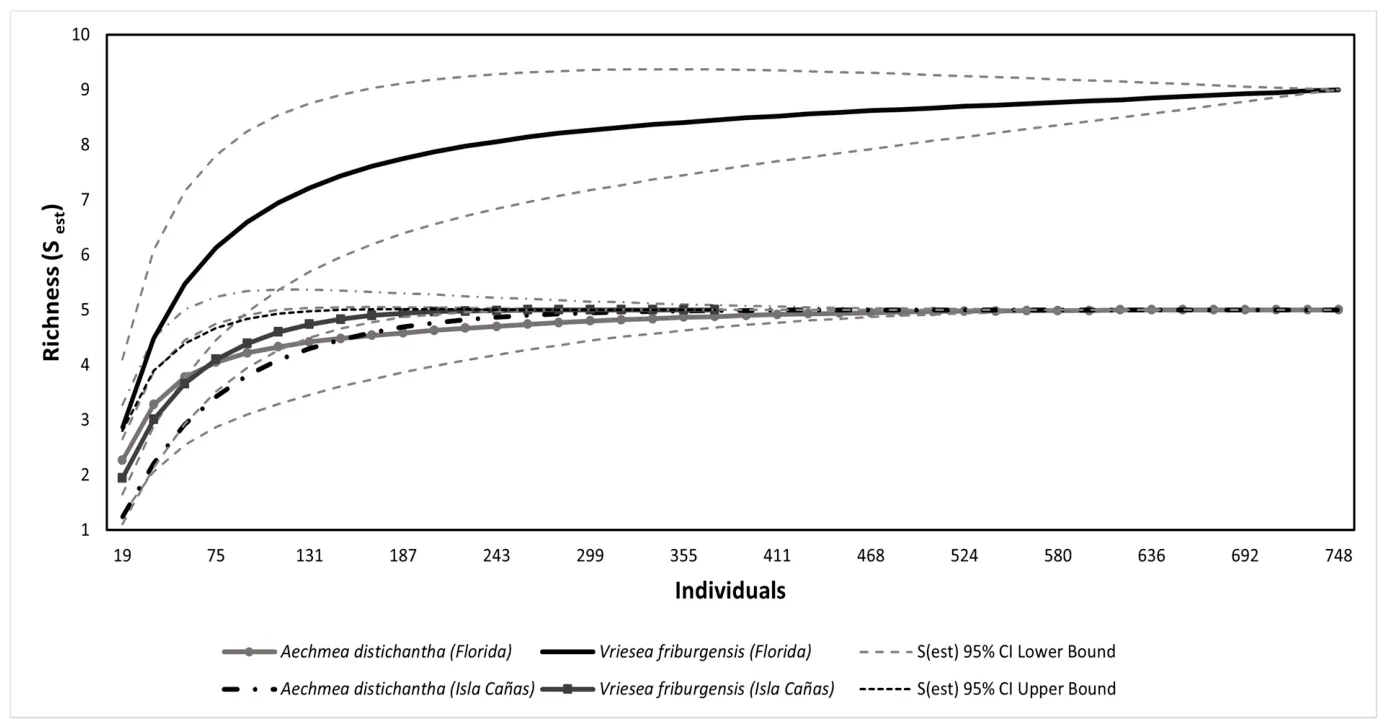
Nonparametric species richness estimators for mosquitoes in bromeliad species in LFPR and Isla de Cañas, north and south (meridional) patches, Yungas region, northwestern Argentina.

The richness observed in Culicidae associated with bromeliads ranged from 5 to 9 species, depending on the locality and bromeliad species. The highest value was recorded in *V*. *friburgensis* in LFPR (Sobs = 9), while 5 species were observed in *A*. *distichantha* in LFPR and in both bromeliads at Isla de Cañas. The ACE, ICE, Chao 1, and Jackknife 1 estimators converged with the observed richness in *A*. *distichantha* in LFPR and in both bromeliads at Isla de Cañas, indicating 100% sampling completeness. Conversely, in *V*. *friburgensis* in LFPR, some estimators were slightly higher than the observed richness (ICE = 9.51; Jackknife 1 = 9.98), with a mean richness of 9.30 species and a sampling completeness of 96.8% (Table 2). These results indicate that the sampling was highly representative across all analyzed combinations; however, for *V*. *friburgensis* in LFPR, the estimations suggest that at least one additional species may have remained undetected. The accumulation and richness estimation curves tended to stabilize as the number of sampled individuals increased. This trend was particularly evident in *A*. *distichantha* in LFPR and in both bromeliads at Isla de Cañas, where the curves rapidly reached an asymptote at approximately 5 species. In the case of *V*. *friburgensis* in LFPR, the curve exhibited higher richness and a more gradual stabilization, approaching 9 species in accordance with the values predicted by nonparametric estimators. Overall, these results demonstrated that the sampling effort was sufficient to capture the majority of the Culicidae richness associated with the studied bromeliads (Figure 3).

### 3.3. Microhabitat Characterization of Bromeliads

The microhabitat variables of *A*. *distichantha* and *V*. *friburgensis* varied across localities, bromeliad species, and sampling periods. The pH levels ranged from 5.139 to 6.067, with the lowest values recorded in *A*. *distichantha* in LFPR and the highest in *V*. *friburgensis* at Isla de las Cañas. Overall, *V*. *friburgensis* exhibited higher pH values than *A*. *distichantha*. Internal water temperature showed marked differences between bromeliad species and localities, ranging from a minimum of 13.55 °C in *A*. *distichantha* at Isla de Cañas in 2017 to a maximum of 23.91 °C in *V*. *friburgensis* in LFPR in 2018. On the whole, the internal temperatures of the bromeliads in LFPR were higher than those recorded at Isla de Cañas, despite interannual and interspecific variations. Height above ground level varied from 148.36 cm to 384.06 cm; the lowest values were recorded in *V*. *friburgensis* in LFPR in 2018, whereas the highest corresponded to *A*. *distichantha* at Isla de Cañas, particularly in 2018. In general, bromeliads at Isla de las Cañas were recorded at a higher height than those in LFPR. Sediment quantity ranged from 0.076 to 2.758, with the highest and lowest values recorded at Isla de Cañas in 2017 in *A*. *distichantha* and *V*. *friburgensis*, respectively. The water volume fluctuated between 206.33 mL and 356.25 mL, with the maximum value in *A*. *distichantha* and the minimum in *V*. *friburgensis*, both recorded at Isla de Cañas in 2017. Taken together, these findings reveal significant environmental heterogeneity among bromeliads, localities, and sampling periods (Table 3).

### 3.4. Bromeliad–Culicidae Interactions

**Figure 4:**
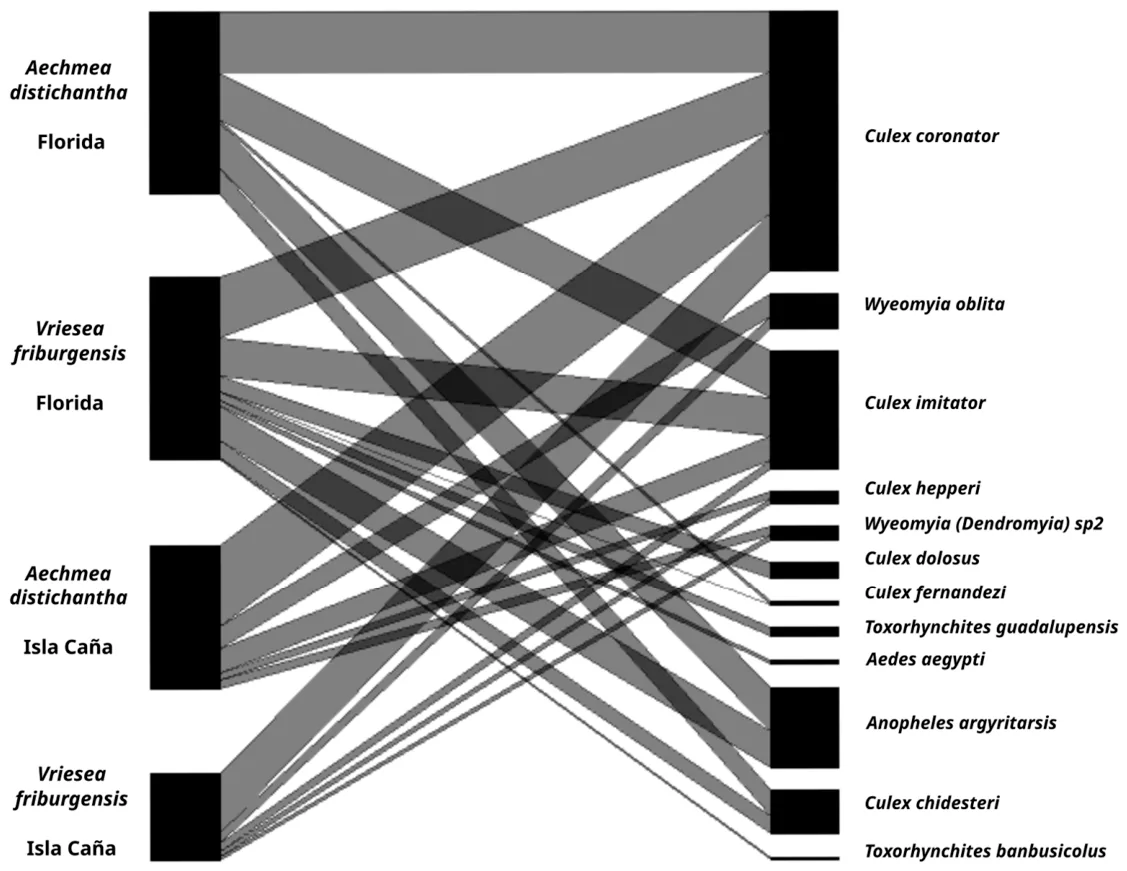
Quantitative bipartite interaction network between bromeliads and Culicidae in LFPR and Isla de Cañas, Yungas region, northwestern Argentina.

The bipartite network constructed from the associations between bromeliads and Culicidae species revealed a connectance of 0.50, indicating that half of the potential interactions between both sets of nodes were observed. The network comprised 12 Culicidae species and 4 bromeliad–locality combinations, forming a single compartment. Furthermore, it exhibited low modularity (Q = 0.188) and low global specialization (H2′ = 0.273), pointing to a relatively generalist interaction pattern. The degree of nestedness was intermediate, with nestedness = 33.05, NODF = 46.94, and weighted NODF = 39.03, suggesting that the less frequent species tended to interact with a subset of the bromeliads used by the most generalist species. The interaction evenness was relatively high (interaction evenness = 0.716; Alatalo interaction evenness = 0.807), reflecting the fact that while some species concentrated a significant portion of the connections, the distribution of interactions was not entirely dominated by a single species. The network also exhibited a moderate niche overlap among Culicidae species (niche overlap HL = 0.428) and high overlap among bromeliads/localities (niche overlap LL = 0.797), indicating that several mosquito species partially shared the same microhabitats, while the assessed bromeliads tended to host relatively similar species assemblages. In accordance with the graphical representation of the network, *Cx*. *coronator* and *Cx*. *imitator* accounted for the majority of the interactions, functioning as the most generalist species in the system, whereas other species, such as *Ae*. *aegypti*, *Cx*. *fernandezi*, *Tx*. *guadalupensis*, and *Tx*. *bambusicola*, exhibited more restricted associations (Figure 4).

### 3.5. Canonical Correspondence Analysis

**Figure 5:**
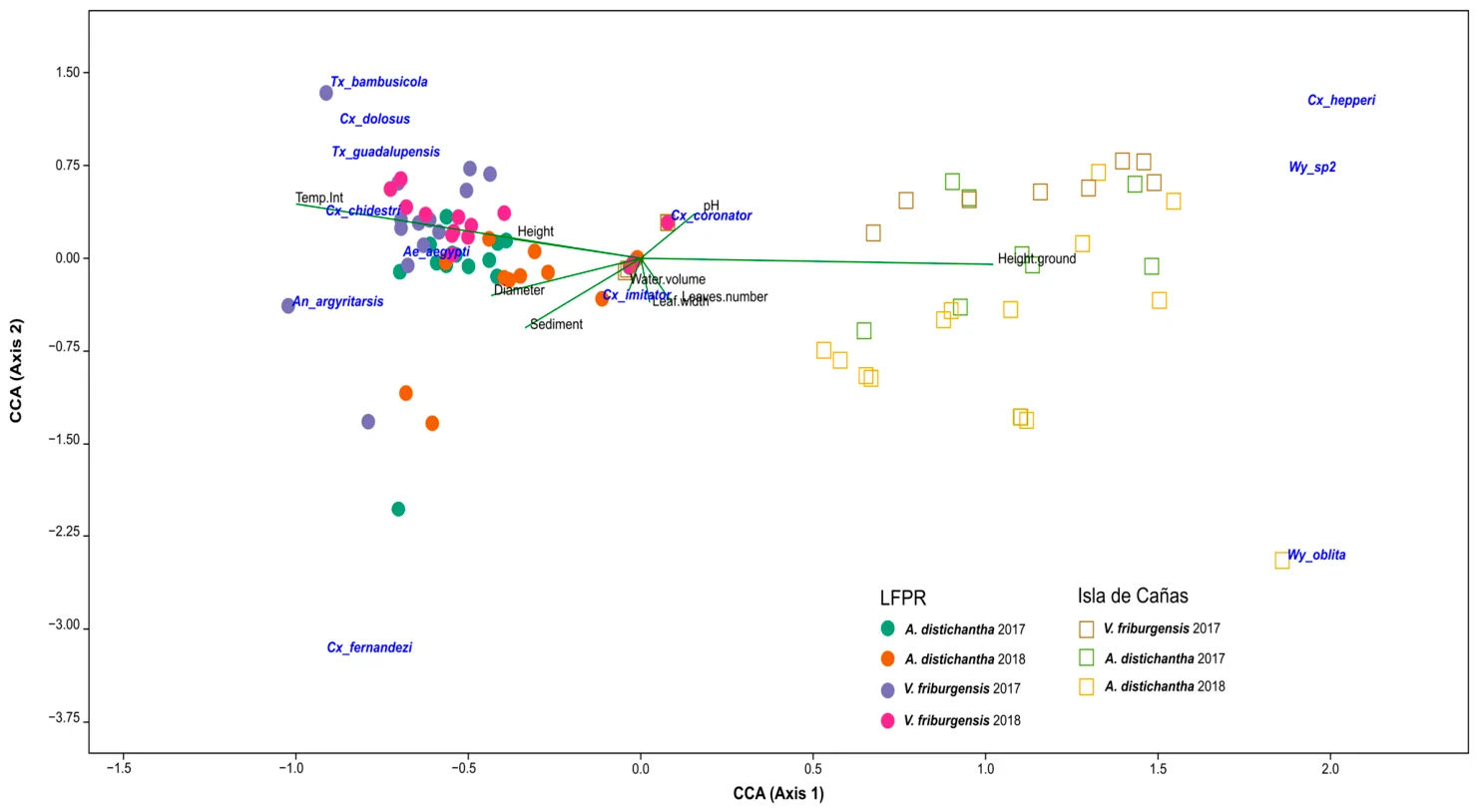
Canonical correspondence analysis (CCA) of Culicidae species composition in relation to bromeliad microhabitat variables. Sampling events across study localities (LFPR and Isla de Cañas, Yungas region, Argentina) are differentiated by host bromeliad species (*Aechmea distichantha* and *Vriesea friburgensis*) and sampling year.

The CCA indicated that Culicidae species composition was structured by environmental variables of the bromeliad microhabitat. Axis 1 had an eigenvalue of 0.3762 and accounted for 63.23% of the constrained inertia, equivalent to 12.72% of the total inertia, being also the only statistically significant axis (*p* = 0.001). Axis 2 presented an eigenvalue of 0.0856 and explained 14.39% of the constrained inertia (2.90% of the total inertia). Together, the first two axes accounted for 77.62% of the variation explained by the environmental variables. Ordination indicated that axis 1 was mainly associated with a positive gradient of height above ground level and a negative gradient of internal temperature, while axis 2 reflected variation related to sediment, internal temperature, and, to a lesser extent, pH. Conversely, variables such as water volume, leaf width, and leaf number showed contributions to sample separation along the primary axes. These results indicate that microhabitat heterogeneity, particularly the position of bromeliad regarding ground level and the internal water temperature, both influenced the local composition of Culicidae. In the ordination space, some species were positioned close to the center of the diagram, suggesting broader associations with different environmental conditions. Notably, *Cx*. *coronator* and *Cx*. *imitator* exhibited scores near the origin of the first axis, in accordance with their widespread occurrence in the studied bromeliads. Conversely, *Wy*. *oblita*, *Cx*. *hepperi*, and *Wy.* sp tended toward positive values of axis 1, whereas *An*. *argyritarsis*, *Cx*. *chidesteri*, *Tx*. *guadalupensis*, *Tx*. *bambusicola*, and *Ae*. *aegypti* tended toward negative or peripheral values, indicating differential associations with the environmental gradients represented by the analysis (Figure 5).

## 4. Discussion

The present study provides evidence regarding the structure of Culicidae communities associated with two bromeliad species across two distinct patches of the Yungas in northwestern Argentina, demonstrating that species composition, diversity, and interactions between mosquitoes and phytotelmata were associated with environmental heterogeneity of the microhabitat. Collectively, our results confirm that bromeliads constitute relevant microecosystems for the Neotropical culicid fauna, not only for hosting high species richness, but also for providing differentiated environmental conditions that favor diverse coexistence patterns [16,17,19,41].This interpretation is consistent with previous reviews and ecological studies highlighting the role of bromeliads as reservoirs of complex aquatic invertebrate communities, including numerous species of Culicidae, some of which are closely associated with this habitat type [4,10,14].

Regarding richness and composition, our results are consistent with prior studies conducted on phytotelmata in northeastern Argentina and the Brazilian Atlantic Forest, where Culicidae diversity varied markedly among host plant species and phytotelmata types [42]. In northeastern Argentina, Álvarez [19] recorded diverse plant–mosquito associations in both native and exotic phytotelmata, highlighting that different plants may sustain assemblages with differential composition. Similarly, studies in Brazil have shown that bromeliads tend to host a diverse mosquito fauna, the structure of which responds to both plant morphology and the physicochemical characteristics of the accumulated water [43].

The greater richness observed in *V. friburgensis* in LFPR suggests that this bromeliad provides a particularly favorable microhabitat for the establishment of a more diverse community of Culicidae. This pattern could be related to differences in plant architecture, water retention capacity, detritus accumulation, and the stability of the aquatic microhabitat, factors previously identified as determinants of richness for organisms associated with bromeliads. Consequently, the differences between bromeliads imply not only changes in the number of individuals but also in the availability of niches and resources for species with distinct ecological requirements.

Thus, Armbruster [44] and Torreias [3] found that various biotic and abiotic factors influence the structure of Culicidae communities in Bromeliaceae, including plant characteristics, leaf number, volume of water retained, and amount of accumulated detritus, which can affect species richness. In this context, Torreias [3] identified a significant correlation between water volume and both the abundance and richness of mosquitoes in the bromeliad *Guzmania brasiliensis* in the Amazon.

In the present work, the total abundance of individuals varied between localities, bromeliad species, and sampling years, with particularly high values recorded in LFPR for both bromeliad species. The observed differences between localities may be related to geographical contrasts [45]. The LFPR is located within a natural environment preserved as a flora and fauna reserve, whereas Isla de Cañas is a small and relatively isolated locality with minimal urban development and minor anthropogenic disturbance [46,47].

The bipartite network analysis revealed a strong dominance of *Cx. coronator* and *Cx. imitator*, which accounted for a significant proportion of the abundances and interactions observed. This pattern suggests that both species exhibit marked ecological plasticity in exploiting different bromelicolous microhabitats [11,48], in contrast to others with more restricted distributions within the network. Nevertheless, both species probably differ in their ecological strategies. *Cx. imitator* is a bromeliad specialist that tends to dominate these microhabitats due to its affinity with the physicochemical conditions of the leaf tanks; it has been reported as one of the dominant species in these microhabitats in different regions of South America, reaching high abundances in *A. distichantha*, *V. friburgensis*, and other Bromeliaceae [9,15,19,25]. In contrast, *Cx. coronator* is a generalist species typically associated with terrestrial–aquatic environments, whose presence in bromeliads might be occasional [17,19].

Regarding dominance and evenness, the mosquito communities associated with *A. distichantha* and *V. friburgensis* presented marked differences between localities and years of sampling. In LFPR, relatively high values of H1 and low values of H2 and evenness were recorded, indicating communities composed of several species but dominated by one or a few that concentrated the greatest abundance of individuals. The availability of phytotelmata with variable environmental characteristics may favor the establishment of species with greater ecological plasticity, capable of exploiting different types of microhabitats within the bromeliad system [4]. Conversely, at Isla de Cañas, higher values of N_2_ and evenness were observed, especially in *A. distichantha* and *V. friburgensis*, suggesting more balanced communities with lower relative dominance. However, the decrease in evenness recorded in *A. distichantha* indicates that the structure of these communities may vary between years, reflecting shifts in the dominance of certain species.

Similar situations have been described in mosquito communities associated with phytotelmata, where some species tend to dominate numerically while others remain at low abundances. In accordance with this pattern, Marques [25] recorded 22 species of Culicidae in bromeliads of the genera *Vriesea* and *Nidularium* in the Brazilian Atlantic Forest, with a marked dominance of *Culex* spp., particularly *Cx. ocellatus*, *Cx. imitator retrosus*, and *Cx. neglectus*. These authors also reported associations between mosquito richness and environmental factors such as the volume of water retained in the phytotelmata and site elevation, observing a greater diversity at lower altitudes. Comparable patterns have been reported in other bromeliad systems, such as the Guapiaçu Ecological Reserve (Brazil), where 14 mosquito species were recorded but with low evenness due to the dominance of *Cx. pleuristriatus*. Similarly, Álvarez [19], in northeastern Argentina for *A. distichantha*, determined Culicidae assemblages with several tribes represented, but with a marked dominance of *Cx. imitator*.

The bipartite network structure also provides a novel component to the study of Culicidae in phytotelmata of Argentina. The combination of moderate connectance, low global specialization, intermediate nestedness, and niche overlap suggests a community where several species partially share the same microhabitats, but where a few generalist species sustain a significant portion of the network architecture [49]. Although studies on Culicidae in Neotropical bromeliads have primarily focused on richness, abundance, distribution, and associations with microenvironmental variables, rather than formal ecological networks, our results align with the general idea that phytotelmata function as systems where coexistence depends on resource heterogeneity and partial habitat partitioning, rather than strict segregation among species [43,50]. In this regard, the coexistence of a bromeliad specialist species like *Cx. imitator* and a generalist species such as *Cx. coronator* reinforces the idea of an ecologically flexible assemblage, in which microhabitat occupancy and relative species abundance are modulated by environmental heterogeneity [9,10].

This pattern has been reported in other systems where species composition and dominance vary as a function of the degree of environmental disturbance. In urban and peri-urban environments of Ilhabela (Brazil), the dominance of species with greater ecological plasticity, such as *Cx. pleuristriatus,* was observed, while species more associated with conserved environments, like *Cx. ocellatus*, presented a lower relative abundance [45]. Likewise, Stein [16] recorded in Tucumán the coexistence of generalist species like *Ae. aegypti* and *Cx. quinquefasciatus* with specialists such as *Cx. imitator* and the predator *Tx. guadeloupensis* in the tanks of *A. distichantha*, evidencing the overlap of ecological strategies in the same microhabitat. In contrast to what has been observed in some conserved systems, where communities may be heavily dominated by bromeliad specialist species, in our study, the network suggests a greater mix of ecological strategies.

The Canonical Correspondence Analysis revealed that environmental gradients modulate the Culicidae community structure in phytotelmata, identifying bromeliad height as the primary factor, followed by internal temperature, and to a lesser extent, sediment and pH. This is consistent with previous studies indicating that the structural characteristics of bromeliads and the physicochemical conditions of the water are determinants in the distribution of mosquitoes that develop in phytotelmata. In the Brazilian Atlantic Forest, it has been documented that the structure of mosquito assemblages is significantly associated with environmental variables such as landscape categories, water volume, and the tank “filling” factor [25]. In particular, those authors observed positive associations between the filling factor and the abundance of *Cx. imitator*, *An. cruzii* and *Cx. neglectus*, which suggests a preference for phytotelmata with greater hydrological stability.

In accordance with the patterns observed in the bipartite networks, species distribution along the environmental gradients defined by the CCA reveals an uneven community structure in which only a subset reaches high abundances. Within this framework, *Cx. coronator* and *Cx. imitator* exhibit differential responses, whereas *Cx. coronator* is associated with the pH and height gradient, evidencing a greater breadth in environmental space; *Cx. imitator* is mainly linked to conditions defined by temperature and sediment. Conversely, species like *Cx. chidesteri*, *Cx. fernandezi*, and *An. argyritarsis* are restricted to specific areas of the environmental space, while taxa such as *Tx. guadeloupensis*, *Tx. bambusicola*, *Cx. dolosus*, and *Ae. aegypti* present less defined associations and a low relative contribution to the community structure. This pattern is consistent with the idea that the microenvironmental heterogeneity of the bromeliads of the Yungas constitutes an important ecological filter for the local structuring of Culicidae assemblages [43].

From a biogeographical perspective, the comparison between LFPR and Isla de Cañas suggests that differences between localities of the Yungas region, likely linked to climatic conditions, vegetation structure, and degree of disturbance, can also be translated into differences in the organization of mosquito communities. Recent studies carried out in Neotropical environments have emphasized that the diversity of available phytotelmata and the surrounding landscape context influence Culicidae composition; consequently, the observed patterns do not depend exclusively on the bromeliad species, but also on the ecological environment in which they are embedded. Therefore, our findings support a multilevel view in which the bromelicolous mosquito community is determined by both the immediate microhabitat and the broader spatial context.

Finally, although the study had an ecological focus, the detection of species such as *Ae. aegypti* in bromeliads highlights the importance of considering phytotelmata not only as biodiversity reservoirs but also as environments potentially relevant from a public health perspective. In the Neotropical literature, it has been noted that certain bromeliads may host vector species or species of medical importance, depending on the landscape, disturbance, and proximity to anthropogenic environments [51].

In summary, our results indicate that the bromeliads of the Yungas sustain ecologically complex Culicidae assemblages, structured by microhabitat heterogeneity and dominated by a combination of generalist species and species of more restricted occurrence. The incorporation of network metrics and ordination analyses demonstrated that these communities differ not only in richness and abundance but also in how species share and exploit phytotelmic microhabitats. This approach contributes to broadening our understanding of Culicidae ecology in Neotropical phytotelmata and provides a useful comparative basis for future studies in environmental gradients and landscapes with different degrees of conservation.

## Figures and Tables

**Table 1:** Abundance, species richness, diversity, and evenness indices of Culicidae associated with *A*. *distichantha* and *V*. *friburgensis* in LFPR and Isla de Cañas, Yungas region, northwestern Argentina.

Subfamily	Tribe	Species	LFPR				Isla de Cañas		
			*A. distichantha*		*V. friburgensis*		*A. distichantha*		*V. friburgensis*
			2017	2018	2017	2018	2017	2018	2017
Anophelinae	Anophelini	*Anopheles* (*Nyssorhynchus*) *argyritarsis*	99	82	103	40			
Culicinae	Aedini	*Aedes* (*Stegomyia*) *aegypti*			19				
	Culicini	*Culex* (*Culex*) *chidesteri*	77	32	30	42			
		*Cx.* (*Cux.*) *coronator*	144	106	135	108	151	186	237
		*Cx.* (*Cux.*) *dolosus*			32	32			
		*Cx.* (*Cux*.) *fernandezi*	9	7	2				
		*Cx.* (*Microculex*) *imitator*	91	101	74	82	19	81	32
		*Cx.* (*Phytotelmatomyia*) *hepperi*					7	20	21
		*Wyeomyia* (*Miamyia*) *oblita*					20	73	47
		*Wy.* sp.					12	23	17
	Toxorhynchitini	*Toxorhynchites* (*Lynchiella*) *guadalupensis*			15	19			
		*Tx.* (*Lyn.*) *bambusicola*			8	2			
		Individuals	420	328	418	325	209	383	354
		Richness (Hill H0)	5	5	9	7	5	5	5
		Hill H1 (eH′)	4.21	4.01	5.89	5.32	2.62	3.76	2.92
		Hill H2 (1/D)	1.34	1.37	1.26	1.28	2.18	1.47	1.92
		Evenness (H1/H0)	0.84	0.8	0.65	0.76	0.52	0.75	0.68

**Table 2:** Nonparametric richness estimators of Culicidae associated with *A*. *distichantha* and *V*. *friburgensis* in LFPR and Isla de Cañas, Yungas region, northwestern Argentina.

Locality	Bromeliad Species	Nonparametric Richness Estimators						Mean	% Sampling Completeness
		S (Observed)	S (Estimated)	ACE	ICE	Chao 1	Jack 1		
LFPR	*A. distichantha*	5	5	5	5	5	5	5	100
	*V. friburgensis*	9	9	9	9.5	9	9.98	9.3	96.8
Isla Cañas	*A. distichantha*	5	5	5	5	5	5	5	100
	*V. friburgensis*	5	5	5	5	5	5	5	100

S = Species, ACE = Abundance-based Coverage Estimator, ICE = Incidence-based Coverage Estimator.

**Table 3:** Microhabitat variables of *A*. *distichantha* and *V*. *friburgensis* in LFPR and Isla de Cañas, Yungas region, northwestern Argentina.

	LFPR				Isla de Cañas		
	*A. distichantha*		*V. friburgensis*		*A. distichantha*		*V. friburgensis*
Variable and SE	2017 (*n* = 18)	2018 (*n* = 15)	2017 (*n* = 16)	2018 (*n* = 14)	2017 (*n* = 12)	2018 (*n* = 16)	2017 (*n* = 15)
pH ^1^	5.139 ^a^	5.233 ^a^	5.906 ^b^	5.964 ^b^	5.708 ^a,b^	5.500 ^a,b^	6.067 ^b^
SE	0.079	0.108	0.050	0.036	0.114	0.112	0.153
Temp. Int (°C) ^2^	21.317 ^a,c,d,e^	20.367 ^a,b,c^	20.169 ^a,c^	23.907 ^a,b,d,e^	13.55 ^f^	17.344 ^g^	22.360 ^a,b,d,e^
SE	0.557	0.273	0.351	0.549	0.157	0.223	0.359
Height from ground (cm) ^3^	156.667 ^a^	163.800 ^a,c^	203.000 ^b^	148.357 ^a^	315.833 ^b,d^	384.063 ^d^	273.000 ^b,c,d^
SE	15.884	13.417	25.289	14.981	30.487	44.119	29.089
Sediment ^4^	1.650 ^a^	1.547 ^a^	0.719 ^a^	1.357 ^a^	2.758 ^a^	1.250 ^a^	0.076 ^b^
SE	0.574	0.279	0.107	0.527	1.058	0.302	0.024
Water volume (mL) ^5^	235.833	281.667	313.125	254.286	356.250	267.500	206.333
SE	28.088	37.946	32.448	37.117	62.320	32.740	19.282

^1^ Kruskal–Wallis H (chi2): 42.31 *p* < 0.001; Mann–Whitney pairwise comparisons with Bonferroni corrections; ^2^ Kruskal–Wallis H (chi2): 76.08 *p* < 0.001; Mann–Whitney pairwise comparisons with Bonferroni corrections; ^3^ Kruskal–Wallis H (chi2): 42.45 *p* < 0.001; Mann–Whitney pairwise comparisons with Bonferroni corrections; ^4^ Kruskal–Wallis H (chi2): 43.3 *p* < 0.001; Mann–Whitney pairwise comparisons with Bonferroni corrections; ^5^ n.s (non-significant). Different superscript lowercase letters within the same row indicate statistically significant differences.

## Data Availability

The data presented in this study are available on request from the corresponding author.

## Abbreviation

The following abbreviation is used in this manuscript:

LFPR	La Florida Provincial Reserve
